# Method-dependent structural evolution of Co_3_O_4_ nanoparticles synthesized *via* sonochemical, chemical precipitation, and hydrothermal routes

**DOI:** 10.1039/d6ra01994c

**Published:** 2026-07-02

**Authors:** Fatma Ismayilova, Zeynab Addayeva, Sevinj Mammadyarova, Eldar Gasimov, Fuad Rzayev, Orkhan Gulahmadov, Mustafa Muradov

**Affiliations:** a Nano Research Laboratory, Baku State University 23 Academik Zahid Khalilov Street 1148 Baku Azerbaijan fatmaismayilova655@gmail.com zeynabaddayeva@gmail.com sevinc.memmedyarova@inbox.ru mbmuradov@gmail.com; b Baku State University, Faculty of Physics, Department of Physics of Semiconductor Azerbaijan; c Department of Cytology, Embryology and Histology, Azerbaijan Medical University. Nasimi Reg. S.Vurgun St., 163 Baku AZ1078 Azerbaijan eldar.gasimov@amu.edu.az; d Electron Microscopy Department, Scientific Research Center, Azerbaijan Medical University. Nasimi Reg. S.Vurgun St., 163 Baku AZ1078 Azerbaijan fuad.rzayev@amu.edu.az; e Faculty of Engineering, Karabakh University AZ2600 Khankendi Azerbaijan orkhan.gulahmadov@karabakh.edu.az

## Abstract

The precise control of microstructural properties in Co_3_O_4_ nanoparticles is crucial for performance, however, the relationship between synthesis method, crystallite size, and lattice strain remains unclear. In this study, Co_3_O_4_ nanoparticles were systematically synthesized using three distinct methods: sonochemical, chemical precipitation, and hydrothermal, to evaluate the method-dependent evolution of their structural characteristics. Phase purity and crystalline structure were rigorously analyzed using X-ray diffraction (XRD), while the Debye–Scherrer equation and the Williamson–Hall method were employed to quantitatively decouple the effects of finite crystallite size and internal microstrain on peak broadening. Key results demonstrate that the synthesis route is a decisive factor in structural tailoring: chemical precipitation yielded the smallest crystallite size (12.1 nm) with the highest dislocation density (6.83 × 10^−3^ nm^−2^), suggesting a defect-rich surface that may enhance catalytic activity. Conversely, the hydrothermal method produced a higher degree of crystallinity with low dislocation density (0.88 × 10^−3^ nm^−2^), but introduced higher residual microstrain (2.49 × 10^−3^) due to rapid growth dynamics. TEM characterization results confirmed the formation of irregular and quasi-spherical morphologies, along with size distribution analysis. UV-Vis spectroscopy revealed two distinct absorption bands corresponding to ligand-to-metal charge transfer (LMCT). A noticeable reduction in the optical band gap was observed for the Co_3_O_4_-CP sample. This shift is directly associated with increased lattice strain identified in structural analysis and quantum confinement effects. SEM and EDS results indicated that the synthesis method strongly affects the morphology of Co_3_O_4_, while confirming the homogeneous presence and distribution of cobalt and oxygen throughout the samples. These findings provide a strategic roadmap for selecting synthesis parameters to engineer Co_3_O_4_ nanostructures with specific defect densities and strain profiles for targeted industrial applications.

## Abstract 2

Co_3_O_4_ nanoparticles show method-dependent crystallinity and morphology; hydrothermal synthesis yields highly crystalline structures, while precipitation produces smaller crystallites.

## Graphical abstract

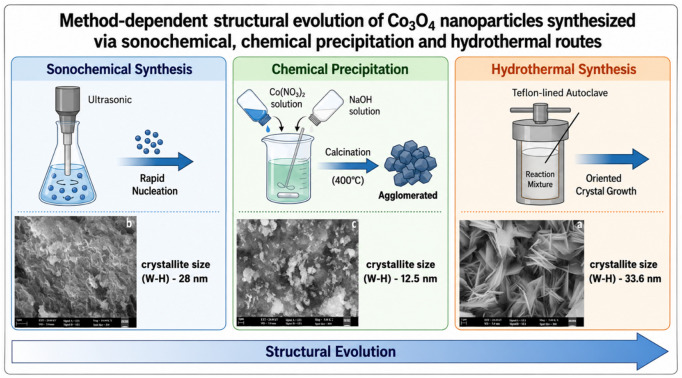

## 1 Introduction

Metal oxide materials form one of the fundamental pillars of modern scientific and technological progress and exhibit a remarkably wide range of application areas. Owing to their high chemical stability, mechanical robustness, and tunable semiconducting properties, these materials play a decisive role in strategic fields such as photocatalysis, energy storage and conversion, gas sensors, magnetic memory devices, lithium-ion batteries, and supercapacitors.^1–3^ In photocatalytic processes, metal oxide nanostructures are indispensable for environmentally oriented applications, including solar-driven water splitting for hydrogen production and the mineralization of organic pollutants.^4^ In the energy sector, these materials significantly enhance the performance of next-generation energy storage devices due to their high specific capacities and excellent cycling stability.^2^ Since the optical, electronic, and magnetic properties of metal oxides are directly governed by their size, morphology, and crystal structure, it becomes essential to refine nanoscale synthesis techniques and to thoroughly investigate how these methods influence the structural evolution of the material.

Among a wide range of functional materials, cobalt oxide (Co_3_O_4_) nanoparticles have attracted considerable research interest.^5,6^ In recent years, the application scope of (Co_3_O_4_)-based materials has significantly expanded, encompassing high-performance supercapacitors, anode materials for lithium-ion batteries, membranes for gas separation, and photocatalysts for wastewater treatment.^7^ Furthermore, emerging studies highlight their potential in the biomedical field, particularly as antibacterial agents and radiosensitizers.^8^ The incorporation of Co_3_O_4_ nanoparticles into triboelectric nanogenerator (TENG) architectures opens new opportunities for surface charge engineering. The coexistence of cobalt cations in multiple oxidation states (Co^2+^ and Co^3+^) facilitates efficient charge carrier trapping, while the introduction of structural defects, such as oxygen vacancies, enables fine tuning of the dielectric properties and surface potential.^9^ Co_3_O_4_ crystallized in a normal spinel structure where Co^2+^ and Co^3+^ ions occupy tetrahedral and octahedral sites, respectively.^10^ This specific cation distribution gives rise to a complex electronic energy structure.

Such electronic transitions are particularly important for TENG applications, as they directly influence the dielectric permittivity and polarization capability of the material under mechanical deformation. The Co–O interatomic bonding plays a crucial role in the formation of the triboelectric response.^11^ The relatively weaker nature of this bond facilitates the migration of oxygen vacancies toward the surface. These vacancies act as charge localization centers and significantly enhance the surface charge density (*σ*) when the material is in contact with polymer matrices such as poly(vinylidene fluoride).^12^ Moreover, studies have demonstrated that hollow nanostructures exhibit a higher specific surface area and promote enhanced polarization of the composite layer due to internal reflection of electric fields.^13^

The selection of a synthesis pathway is a critical factor in determining the structural characteristics and functional efficiency of Co_3_O_4_ nanomaterials. Sonochemical synthesis leverages the phenomenon of acoustic cavitation induced by high-intensity ultrasonic waves in liquid media.^14,15^ The violent collapse of cavitation bubbles generates localized “hot spots” with extreme conditions-temperatures of approximately 5000 K and pressures up to 1000 atm which facilitate rapid precursor decomposition and nucleation.^16^ The exceptionally high cooling rates inherent in this process suppress excessive crystal growth, yielding small, narrowly distributed nanoparticles; for instance, optimized sonication parameters can produce Co_3_O_4_ crystals as small as 10–15 nm.^17,18^

In contrast, hydrothermal synthesis is conducted within closed autoclaves at temperatures exceeding the boiling point of water. This high-pressure environment alters the dielectric constant and viscosity of water, significantly enhancing the solubility and chemical reactivity of precursors. A primary advantage of this technique is the precise control over morphology enabling the fabrication of nanorods, nanotubes, and mesoporous spheres through the adjustment of pH, temperature, and template agents.^19^ While the hydrothermal method ensures superior crystallinity and phase purity for the Co_3_O_4_ spinel structure, its widespread application is often hindered by long reaction times and the necessity for subsequent calcination.^20,21^ Conversely, the chemical precipitation method remains the industrial preference due to its cost-effectiveness and scalability, despite often resulting in lower crystallinity, higher lattice distortions, and a greater tendency for particle agglomeration.^22^

Recent studies demonstrate that Co_3_O_4_ samples synthesized at low temperatures (*e.g.*, 260 °C) exhibit highly defect-rich structures. These defects are mainly cobalt vacancies (V_Co_), which lead to deviations in stoichiometry toward Co_3_O_4_-δ. With increasing temperature, the concentration of these vacancies decreases, and the nominal stoichiometry (O/Co = 4/3) is restored. Sonochemical and hydrothermal methods provide broader opportunities for such defect engineering.^23^ Furthermore, depending on the anion of the precursor salt, different stoichiometries of α-Co(OH)_2_ precursors can be obtained, which is reflected in the color of the final product (pink or blue). Calcination of these precursors at 260 °C results in the formation of pure cubic spinel-phase Co_3_O_4_. Studies also show that low concentration salt solutions favor the formation of nanoplates enriched in octahedrally coordinated Co^2+^ ions, whereas higher concentrations promote the formation of nanostructures containing both octahedral and tetrahedral cobalt coordination sites.^24^

As can be seen from Table 1, the sonochemical method and the low-temperature sol–gel process yield the smallest crystallite sizes (15–20 nm), which consequently result in the highest dislocation density and microstrain values.^25^ This behavior can be attributed to the rapid quenching and intense energy transfer occurring during acoustic cavitation, which introduce a high concentration of lattice defects and internal stresses within the crystal structure. In contrast, the hydrothermal and high-temperature sol–gel routes favor the formation of significantly larger crystallites (47–85 nm).^29^ The pronounced reduction in internal strain and dislocation density observed for larger crystallites indicates an improvement in crystal quality, associated with enhanced atomic rearrangement toward thermodynamically stable lattice positions. Regarding the lattice parameter, samples synthesized *via* hydrothermal and sonochemical methods exhibit values closer to that of bulk Co_3_O_4_ (8.08 Å), whereas lattice contraction is observed in precipitation-based and low-temperature synthesis routes. This contraction is commonly linked to surface stress effects and non-stoichiometric oxygen distribution within the crystal lattice.^6^ Consequently, for specific technological applications-whether those requiring high surface area, such as photocatalysis, or those demanding high crystallinity, such as sensor devices the choice of synthesis method emerges as the most critical factor governing the structural evolution of the material.^9^ Despite extensive research, existing literature predominantly examines Co_3_O_4_ nanoparticle synthesis through isolated lenses, often lacking a comparative framework for quantitative microstructural analysis across diverse methods.^25^ As a result, the fundamental relationship between the synthesis pathway and the induced microstructural features-such as crystallite size, microstrain, and dislocation density has not been fully elucidated for Co_3_O_4_ samples with identical chemical compositions. While most contemporary studies primarily focus on morphological control, the present work distinguishes itself by providing a rigorous analysis of the correlation between synthesis-induced lattice strain, dislocation density, and phase purity of the spinel lattice. In addition, most existing studies focus on the application areas of materials; however, the mechanisms governing the control of intrinsic lattice strain and structural defects during the synthesis process have largely been overlooked. The novelty of this study lies in the first comprehensive comparative analysis of the distinct microstructural evolution induced by three different synthesis methods (hydrothermal, sonochemical, and chemical precipitation) under identical thermal conditions. The main scientific contribution of this work is the identification of the “fingerprints” of the synthesis method on both the structural and optical properties of the material, as well as the fundamental elucidation of the role of internal microstrain in tuning the band gap.

**Table 1:** Comparison of structural parameters depending on the synthesis method

Synthesis method	Crystallite size (*D*, nm)	Microstrain (*ε*, ×10^−3^)	Dislocation density *δ* (×10^−3^) nm^−2^	Lattice parameter *a*, (Å)	References
Sonochemical	15.78	5.23	2.11	8.080	25
Chemical precipitation	25.62	2.10	15.20	7.970	26
Chemical precipitation	28.37	1.1–1.5	1.24	8.0816	27
Hidrothermal	25.0–85.0	1.20	1.38–16.00	8.080	19
Hidrothermal	30.6	—	—	8.084	18
Sol–gel	16.20	7.75	38.10	7.948	28
Sol–gel	47.0–67.0	1.10–1.80	0.22–0.45	8.071	29
Sol–gel	20.10	6.73	7.10	8.062	30

## 2 Experimental

### 2.1. Materials and reagents

All chemical reagents used in this study were of analytical grade and used as received without further purification. Cobalt(ii) nitrate hexahydrate (Co(NO_3_)_2_·6H_2_O), urea (CO(NH_2_)_2_), polyvinyl alcohol (PVA, MW = 89 000 g mol^−1^), and potassium hydroxide (KOH) were purchased from Sigma-Aldrich.

### 2.2. Synthesis of the Co_3_O_4_ nanoparticles

#### 2.2.1. Preparation of the Co_3_O_4_ NPs *via* hydrothermal method (Co_3_O_4_-HT)

**Fig. 1:**
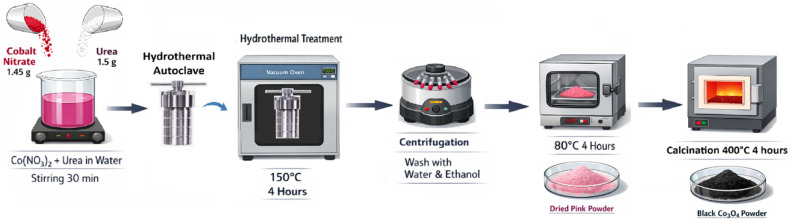
Schematic representation of Co_3_O_4_ nanoparticles synthesis by hydrothermal method.

Co_3_O_4_ nanoparticles were synthesized *via* a hydrothermal method.^31^ Initially, 1.45 g of cobalt nitrate hexahydrate (Co(NO_3_)_2_·6H_2_O) and 1.5 g of urea were dissolved in 40 mL of distilled water and stirred at room temperature on a magnetic stirrer for 30 min until a homogeneous solution was obtained. The resulting solution was transferred into a 100 mL Teflon-lined stainless steel autoclave. The autoclave was tightly sealed and maintained at 150 °C (approx. 4.5–5 bar at 150 °C, with a slight increase expected due to urea decomposition) in an oven for 4 h. After completion of the reaction, the autoclave was allowed to cool naturally to room temperature. The obtained pink precipitate was washed several times with distilled water and ethanol to remove unreacted ions and organic residues. Subsequently, the sample was dried at 80 °C for 4 h in a vacuum oven. The dried precursor phase was calcined in a muffle furnace at 400 °C for 4 h to form the crystalline Co_3_O_4_ phase. As a result, black-colored Co_3_O_4_ nanoparticles were obtained (Fig. 1).

#### 2.2.2. Preparation of the Co_3_O_4_ NPs *via* sonochemical method (Co_3_O_4_-SC)

**Fig. 2:**
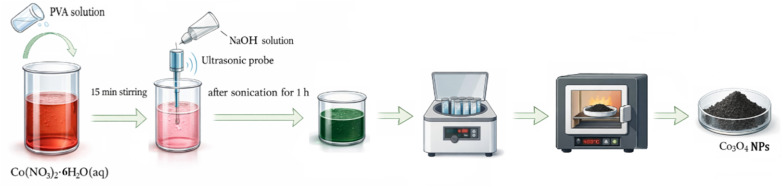
Schematic representation of Co_3_O_4_ nanoparticles synthesis by sonochemical.

Co_3_O_4_ nanoparticles were synthesized *via* a sonochemical method.^32^ Initially, 3.09 g of cobalt nitrate hexahydrate (Co(NO_3_)_2_·6H_2_O) was dissolved in 100 mL of distilled water. Separately, 1 g of polyvinyl alcohol (PVA) was dissolved in 50 mL of distilled water and subsequently added to the cobalt nitrate solution. The resulting mixture was stirred at room temperature on a magnetic stirrer for 30 minutes until a homogeneous solution was obtained. Then, 0.8 g of KOH was dissolved in 100 mL of distilled water to prepare an alkaline solution, which was dropwise added to the Co(NO_3_)_2_-PVA solution under ultrasonication (20 kHz, 500 W). The mixture was further sonicated for 1 hour. The resulting dark green precipitate was separated by centrifugation and washed twice with distilled water. The obtained powder was then poured into a Petri dish and dried at room temperature. Finally, the dried precursor was calcined at 400 °C for 4 hours in a muffle furnace to form the crystalline Co_3_O_4_ phase. As a result, black Co_3_O_4_ nanoparticles were obtained (Fig. 2).

#### 2.2.3. Preparation of the Co_3_O_4_ NPs *via* chemical precipitation method (Co_3_O_4_-CP)

**Fig. 3:**
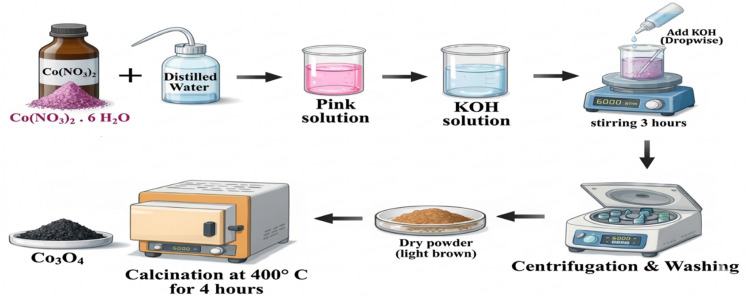
Schematic representation of Co_3_O_4_ nanoparticles synthesis by chemical precipitation.

Co_3_O_4_ nanoparticles were synthesized *via* a chemical precipitation method.^22^ Initially, 0.58 g of cobalt nitrate hexahydrate (Co(NO_3_)_2_·6H_2_O) was dissolved in 20 mL of distilled water. Separately, 0.22 g of KOH was dissolved in 20 mL of distilled water and added dropwise to the cobalt nitrate solution, then stirred at room temperature on a magnetic stirrer for 3 hours. The resulting brown precipitate was separated by centrifugation and washed several times with distilled water and ethanol. The obtained powder was then transferred to a Petri dish and dried at room temperature. To induce the formation of the crystalline Co_3_O_4_ phase, the dried precursor was calcined in a muffle furnace at 400 °C for 4 hours. As a result, black-colored Co_3_O_4_ nanoparticles were obtained. All synthesized Co_3_O_4_ samples were subjected to an identical post-synthesis calcination procedure to allow unbiased comparison of structural properties. The calcination was performed in air at 400 °C for 4 h with a heating rate of 10 °C min^−1^ for all samples. The samples obtained *via* hydrothermal, sonochemical, and chemical precipitation methods are hereafter referred to as Co_3_O_4_-HT, Co_3_O_4_-SC and Co_3_O_4_-CP, respectively (Fig. 3).

### 2.3. Instrumentation and structural data measurements

A bath-type ultrasonicator (VCX500) was used for the synthesis of the Co_3_O_4_-SC sample. X-ray diffraction (XRD) measurements were performed using a Bruker D8 Endeavor diffractometer with nickel-filtered Cu Kα radiation (*λ* = 1.5406 Å). Data were collected with a step size of 0.1° and a scan rate of 5° 2*θ*/min over a 2*θ* range of 10° to 90°, ensuring adequate angular resolution and well-defined diffraction peaks. The optical properties were analyzed using a Specord 250 Plus UV-Vis Spectrophotometer over a wavelength range of 190–1100 nm. The morphology of the samples was examined with a JEM-1400 Transmission Electron Microscope (JEOL, Japan) operated at an accelerating voltage of 80–120 kV. The morphology of the samples were investigated using a Hitachi S–3400N Scanning Electron Microscope (SEM) equipped with an energy-dispersive X-ray spectroscopy (EDS) system for elemental analysis.

## 3 Results and discussion

### 3.1. The formation mechanisms of Co_3_O_4_ nanoparticles

The preparation of Co_3_O_4_ nanoparticles *via* sonochemical, chemical precipitation, and hydrothermal methods, despite differing in reaction conditions, involves similar fundamental physicochemical steps: dissolution of the initial metal ions, formation of the precursor phase, drying, and subsequent thermal calcination to obtain spinel-structured Co_3_O_4_. Initially, in all these methods, cobalt nitrate hexahydrate (Co(NO_3_)_2_·6H_2_O) is dissolved in water, resulting in the presence of Co^2+^ ions in the solution.Co(NO_3_)_2_·6H_2_O → Co^2+^ + 2NO^3−^ + 6H_2_O

The subsequently added alkaline reagent (*e.g.*, KOH) neutralizes the hydrogen ions in the solution, leading to the formation of an amorphous Co(OH)_2_ precipitate:Co^2+^ + 2OH^−^ → Co(OH)_2_↓

This precursor precipitate is formed *via* the reaction of Co^2+^ ions with hydroxide ions and serves as the primary precursor phase in all methods. In the sonochemical approach, the cavitation effects generated by ultrasonication locally increase the temperature and pressure in the solution, facilitating the formation of smoother, finer, and more homogeneous particles. Simultaneously, stabilizers such as polyvinyl alcohol (PVA) complex Co^2+^ ions with polymer chains, ensuring uniform particle distribution and preventing aggregation.^33^ In hydrothermal solutions, urea (CO(NH_2_)_2_) undergoes slow hydrolysis at elevated temperatures, generating ions such as OH^−^ and carbonate (CO_3_^2−^). These ions lead to a gradual and controlled increase in pH in the solution, promoting the hydration of cobalt(ii) ions. Thus, urea acts as a source of OH^−^ and CO_3_^2−^ ions *via* hydrolysis. The OH^−^ ions react with cobalt(ii) ions to form cobalt(ii) hydroxide and carbonate complexes. Since this process occurs gradually under hydrothermal conditions, it allows the formation of crystalline precursor structures of the sample.^34^CO(NH_2_)_2_ + 3H_2_O → 2NH_4_^+^ + CO_3_^2−^ + 2OH^−^In all these methods, after the precursor phase is dried, the temperature is increased, and the transition from the hydroxide/carbonate precursor to the oxide phase with spinel-structured Co_3_O_4_ occurs *via* thermal calcination. At this stage, the dehydration and oxidation reaction mechanisms are manifested, forming the spinel structure that involves both Co^2+^ and Co^3+^ ions. The generalized reaction scheme is as follows:3Co(OH)_2_ + 1/2O_2_↑ → Co_3_O_4_ + 3H_2_O↑

As a result, cubic spinel-structured Co_3_O_4_ nanoparticles are obtained; the final product exhibits high crystallinity, since during the thermal treatment, free water is removed from the precursors and oxidation is rapidly completed. In the sonochemical method, PVA and other organic compounds are completely decomposed at this stage and released from the system in the gaseous phase.

### 3.2. Characterization of Co_3_O_4_ nanoparticles

**Fig. 4:**
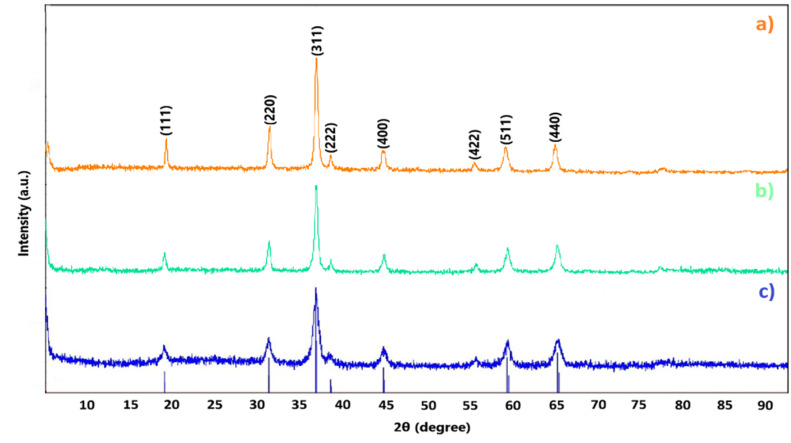
XRD patterns of (a) Co_3_O_4_-HT, (b) Co_3_O_4_-SC and (c) Co_3_O_4_-CP.

The crystal phases of Co_3_O_4_ samples obtained *via* various synthesis methods were characterized using XRD. The XRD patterns of Co_3_O_4_ nanoparticles are presented in Fig. 4. Several characteristic diffraction peaks were observed at different diffraction angles in the XRD spectra. The diffraction peaks recorded at 2*θ* = 19.1°, 31.4°, 37.1°, 38.7°, 44.8°, 55.7°, 59.4°, and 65.2° correspond to the (111), (220), (311), (222), (400), (422), (511), and (440) crystal planes, respectively, confirming the presence of a face-centered cubic (fcc) spinel Co_3_O_4_ structure, no impurity phases (such as CoO or metallic Co) were detected, confirming the high phase purity of the synthesized spinel oxide (ICDD Card No. 42-1467).^35^ The detected crystal planes are consistent with the typical cubic spinel structure of Co_3_O_4_, in which Co^2+^ ions occupy tetrahedral sites, while Co^3+^ ions occupy octahedral sites. The sharp and intense diffraction peaks observed in the XRD patterns indicate that the synthesized samples possess a high degree of crystallinity. Moreover, the absence of additional peaks corresponding to secondary phases.

### 3.3. Average crystallite size and lattice strain

#### 3.3.1. Debye–Scherrer's method

XRD peak profile analysis is regarded as one of the most straightforward yet powerful approaches for evaluating peak broadening associated with crystallite size reduction and lattice strain originating from dislocations and other crystal defects. The observed broadening of XRD diffraction peaks arises from a combination of instrumental effects and intrinsic sample-related contributions. In order to accurately distinguish and quantify the individual contributions of crystallite size and lattice strain, it is essential to eliminate the instrumental broadening component from the measured peak width. For Co_3_O_4_, the instrument-corrected broadening corresponding to each diffraction plane (*hkl*), denoted as *β*_*hkl*_, can be estimated using the following relation.^36^1
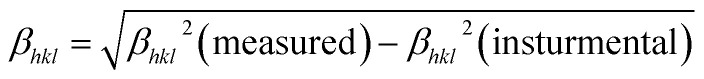


Notably, the noticeably higher intensity of the (311) diffraction peak for Co_3_O_4_ compared to the other reflections indicates that the as-synthesized Co_3_O_4_ nanocrystals exhibit a preferred crystallographic orientation along the (311) plane. This preferential growth suggests that a larger fraction of crystallites is aligned in the direction corresponding to the interplanar spacing (*d*) associated with specific Miller indices (*hkl* = 311). The degree of preferential orientation can be quantitatively evaluated using the following expression:

2 *nλ* = 2*d* sin *θ*

In this context, *n* denotes the order of diffraction (scattering), which is typically taken as *n* = 1, while *θ* represents the Bragg angle. For a cubic crystal system, the interplanar spacing (*d*-spacing) can be expressed as a function of the lattice constant and the corresponding Miller indices (*hkl*). The crystallographic planes identified from the XRD patterns, along with their associated diffraction angles (2*θ*), were employed to determine the lattice parameters of the cubic unit cell using a nonlinear fitting approach based on eqn (3). For the cubic structure under consideration, the lattice constants were assumed to be equal (*a* = *b* = *c*, in Å), and the calculated values are summarized in Table 2.3
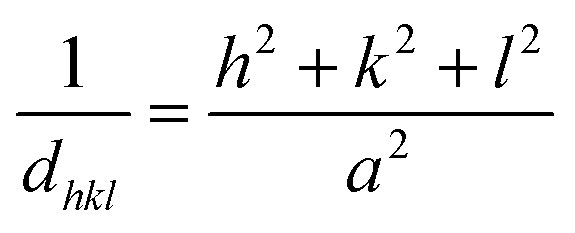
For Co_3_O_4_ nanoparticle samples synthesized *via* all three preparation routes, the average crystallite size (*D*) was estimated from the most intense diffraction peaks using the Debye–Scherrer approach, according to the following equation.^37^4
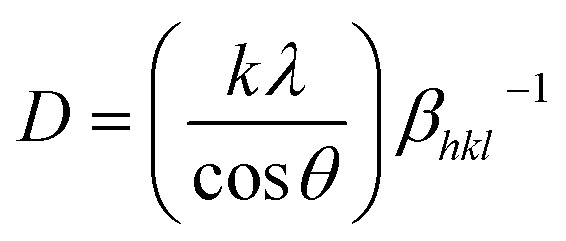
Here *θ* represents the glancing (Bragg) angle, *k* = 0, 9 denotes the shape factor, *λ* corresponds to the wavelength of CuKα radiation, and *β*_*hkl*_ refers to the broadening of the diffraction peak. The degree of structural imperfections present in the samples can be evaluated through the dislocation density 
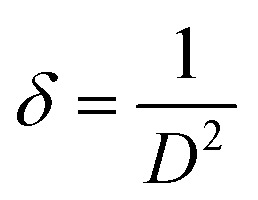
 which is defined as the total length of dislocation lines per unit volume of the crystal.^38^ It is well established that the calculated *δ* value is closely associated with the concentration of defects formed during the growth of Co_3_O_4_ crystallites. Accordingly, the dislocation density was determined for all three samples (Co_3_O_4_-HT, Co_3_O_4_-SC, and Co_3_O_4_-CP), and the obtained values are summarized in Table 2.

**Table 2:** XRD analysis and lattice parameter determination of Co_3_O_4_ nanoparticles synthesized *via* three different methods

Samples	Synthesis methods	*T*_calc_ (°C)	*D* Average crystallite size (nm)			Microstrain (×10^−3^) (calculated by Williamson–Hall method)	Lattice parameters for the cubic structure *a* = *b* = *c*, (Å)	Dislocation density, δ (×10^−3^) line nm^−2^
			Debye–Scherrer method	Williamson–Hall method	Modified Scherrer			
Co_3_O_4_ -HT	Hydrothermal	400	18.75	33.6	21.82	2.49	8.03	0.88
Co_3_O_4_ -SC	Sonochemical	400	19.18	28	22.13	1.73	8.05	1.28
Co_3_O_4_ -CP	Chemical precipitation	400	12.1	12.5	11.63	0.47	8.07	6.38

### 3.4. Evaluation of microstrain

#### 3.4.1. Williamson–Hall methods

Crystallite size estimation is not limited solely to the Debye–Scherrer approach; therefore, the Williamson–Hall (W–H) analysis was additionally employed to account for peak broadening effects arising from both crystallite size dispersion and microstrain induced by lattice imperfections. In powdered materials, the broadening of diffraction peaks may originate from lattice distortions and structural disorder caused by defects and dislocations between atomic planes. The strain-induced contribution to peak broadening resulting from such lattice deformations can be expressed by the following relationship.^39^5
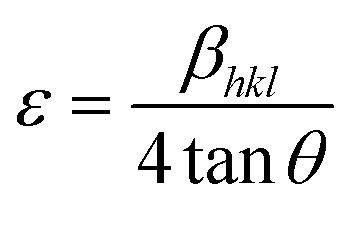


Based on eqn (4) and (5), it can be inferred that the diffraction peak broadening originating from the finite crystallite size exhibits an inverse dependence on the cos(*θ*) function, whereas the broadening induced by lattice strain shows an inverse relationship with the tan(*θ*) function. Consequently, the total observed peak broadening arises from the combined contribution of crystallite size effects and microstrain present within the sample, and can be expressed as follows.^40,41^6
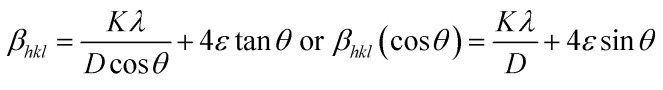


The average crystallite sizes and the corresponding microstrain values determined using both methods are summarized in Table 2.

Table 2 summarizes the crystallite sizes calculated using the Debye–Scherrer and Williamson–Hall (W–H) methods. The results show that the chemical precipitation method yields the smallest crystallite size, which is 12.5 nm according to the W–H method. In contrast, the hydrothermal synthesis leads to the largest crystallite size, reaching 33.6 nm as estimated by the W–H approach. The discrepancy between the crystallite sizes obtained from the Scherrer and W–H methods can be attributed to the ability of the W–H model to account for lattice strain in addition to size-induced peak broadening. The hydrothermally synthesized sample exhibits the highest microstrain value (2.49) along with a relatively high dislocation density, suggesting that crystal growth under elevated temperature and pressure promotes the formation of internal lattice defects. Interestingly, despite possessing the smallest crystallite size, the Co_3_O_4_-CP sample shows the lowest microstrain value (0.47). However, this sample demonstrates a significantly higher dislocation density (6.83 × 10^−3^ nm^−2^) compared to the other samples. For all synthesized samples, the calculated lattice parameter (*a*) lies within the range of 8.03–8.07 Å, which closely matches the standard value reported for cubic spinel Co_3_O_4_ (ICDD No. 42-1467).^35^ The slight lattice expansion observed in the chemically precipitated sample (8.07 Å) can be associated with surface effects and altered ionic arrangements in nanoscale crystallites.^42^ Since the dislocation density (*δ*) is inversely proportional to the square of the crystallite size (*δ* = 1/*D*^2^), the smallest crystallite size observed for the Co_3_O_4_-CP sample results in an increased number of grain boundaries and dislocations per unit area. This structural characteristic is advantageous for catalytic applications, as it provides a higher density of active sites.^43^ The observed higher dislocation density suggests a potential for enhanced catalytic activity, as lattice defects often serve as active sites for surface reactions, consistent with recent studies demonstrating a positive correlation between defect density and catalytic performance.^44^ However, further experimental catalytic validation is required to confirm this potential. Both sonochemical and hydrothermal synthesis methods introduce substantial energy into the system-via ultrasonic cavitation and thermal energy, respectively-thereby accelerating crystal growth and inducing higher levels of internal lattice strain. The slight deviation of the lattice parameter from the bulk value of 8.08 Å can be explained by the reduced coordination number of surface atoms in nanoparticles, leading to the phenomenon commonly referred to as lattice expansion.^7^

**Fig. 5:**
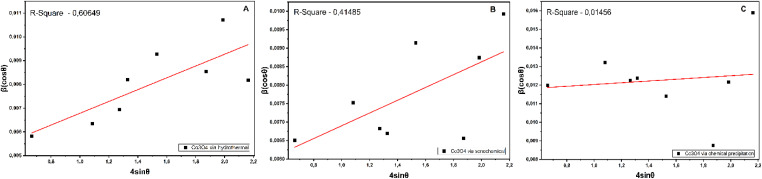
Williamson–Hall plots and linear regression analysis of the extracted data for (A) Co_3_O_4_-HT, (B) Co_3_O_4_-SC, and (C) Co_3_O_4_-CP nanoparticle samples.

Fig. 5 presents the Williamson–Hall (W–H) analysis results for each synthesized sample. The estimated crystallite sizes of all three samples (Co_3_O_4_-HT, Co_3_O_4_-SC, and Co_3_O_4_-CP) fall within the range of approximately 12–33 nm, revealing noticeable variations among the samples. The linear fitting of the W–H plots indicates that the slopes are positive for all specimens. According to the Williamson–Hall approach, a positive slope is indicative of the presence of tensile lattice strain within the synthesized Co_3_O_4_ nanostructures.^45^ Such strain is commonly attributed to the formation of lattice imperfections during nanoparticle growth, including vacancies, surface-related defects, and deviations of atoms from their equilibrium positions. Notably, the Co_3_O_4_-HT sample exhibits the steepest positive slope, suggesting that the higher thermal energy employed during this synthesis route induces a more pronounced lattice expansion and consequently higher tensile strain. This positive slope further implies that the internal stress within the crystal lattice is directed toward the particle boundaries, *a* factor that can significantly influence the optical and magnetic properties of the material.^46–50^ In contrast, the sonochemically synthesized Co_3_O_4_-SC sample (Fig. 5B) displays a moderate level of microstrain, which can be attributed to the rapid nucleation and defect generation promoted by acoustic cavitation effects inherent to the sonochemical process. Meanwhile, the chemically precipitated Co_3_O_4_-CP sample (Fig. 5C) shows the lowest slope value, indicating reduced lattice distortion and the formation of a comparatively more relaxed crystal structure. Despite the observed differences in microstrain, the crystallite sizes calculated from the *y*-intercepts of the W–H plots remain within the nanometer scale for all samples, confirming the successful synthesis of nanosized Co_3_O_4_. Overall, these findings demonstrate that the synthesis method plays a decisive role in tailoring the internal lattice strain and defect density of Co_3_O_4_ nanoparticles, parameters that can critically affect their functional properties.

**Fig. 6:**
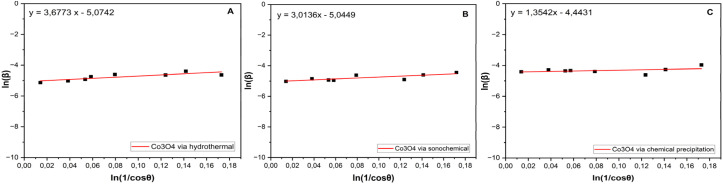
The modified Scherrer plots of (A) Co_3_O_4_-HT, (B) Co_3_O_4_-SC, and (C) Co_3_O_4_-CP nanoparticle samples.

Looking closer at the crystals and how much strain they carry, scientists used a tweaked version of the Scherrer method.^51^ In Fig. 6, three types of samples are shown made by hydrothermal, sonochemical, and chemical precipitation-each plotted as ln *β* against ln(1/cos *θ*) This way differs from the old formula because it reduces mistakes from uneven particle sizes and equipment flaws. It gives a better idea of how big the crystals are, labeled here as *D*. A different approach relies on the logarithmic link tied to the formula ln *β* = ln(1/cos *θ*) + ln(*kλ*/*D*) where *β* stands for the full width at half maximum (FWHM) of the XRD peaks, *k* is the shape factor (0.94), and *λ* is the X-ray wavelength. Looking at the graphs, each method's data points follow a straight path clearly, supported by solid correlation values. From these lines, the crystal size can be worked out simply by reading the *y*-intercept. Sample A, made *via* hydrothermal process, gives a line described as *y* = 3.6773*x* − 5.0742. That steeper slope hints at differences in how atoms are arranged – possibly irregular spacing or disorder shaping its behavior. B comes from sonochemistry, its pattern fits *y* = 3.0136*x* − 5.0449, showing ultrasonic pushes formed medium-sized crystals with steady lattices. On the flip side, sample C made by regular chemical reaction has a slope just 1.3542 – hinting at tiny particles where crystals match well and carry minimal strain inside. Together, findings using the tweaked Scherrer technique prove how making method shapes both particle size and hidden lattice flaws in Co_3_O_4_ bits. Values pulled straight from intercepts stand strong as proof of structure quality, fitting neatly beside outputs found through classic Scherrer approach.

**Fig. 7:**
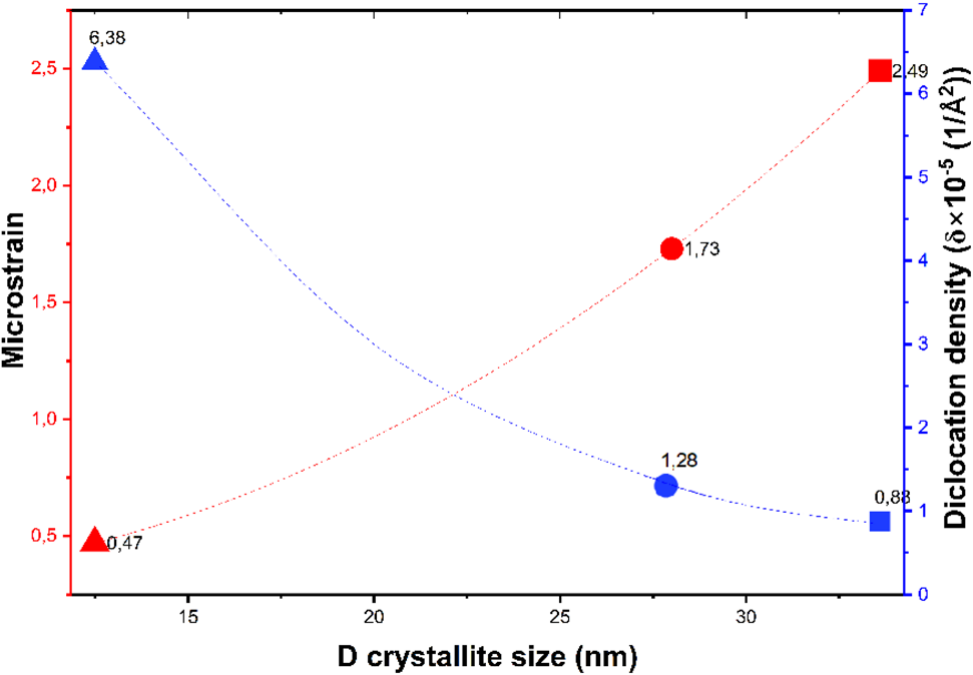
The correlation between crystallite size, microstrain, and dislocation density for Co_3_O_4_-HT (■ square), Co_3_O_4_-SC (● circle), and Co_3_O_4_-CP (▲ triangle) nanoparticles, as determined by the Williamson–Hall method.

The interdependence of the quantitative parameters obtained from the Williamson–Hall analysis is summarized in Fig. 7. As clearly evidenced by the plot, an inverse correlation exists between the crystallite size (*D*) and both the microstrain (*ε*) and the dislocation density (*δ*). With increasing crystallite size (movement toward the right along the *x*-axis), a noticeable increase in microstrain is observed, particularly for the Co_3_O_4_-CP sample. This behavior can be attributed to the accumulation of lattice distortions during the formation of larger crystallites, leading to enhanced internal strain within the crystal lattice. In contrast, the dislocation density (*δ*) exhibits a pronounced increase with decreasing crystallite size. The Co_3_O_4_-SC sample shows the highest dislocation density value (6.83 × 10^−3^ nm^−2^), which can be explained by the higher density of grain boundaries typically present in smaller crystallites. The trends illustrated in Fig. 7 confirm that nanoparticles synthesized *via* the chemical precipitation route tend to possess a higher concentration of structural defects, particularly dislocations, whereas the hydrothermal method promotes the growth of larger crystallites accompanied by elevated internal microstrain. This correlation visually demonstrates how the synthesis approach governs not only the particle morphology but also the internal atomic arrangement of the material. Specifically, the downward trend of the blue curve (dislocation density) with increasing crystallite size and the simultaneous upward trend of the red curve (microstrain) indicate that crystallite growth leads to a reduction in surface-related defects, such as dislocations, while intensifying strain within the crystal volume.

### 3.5. TEM analysis

**Fig. 8:**
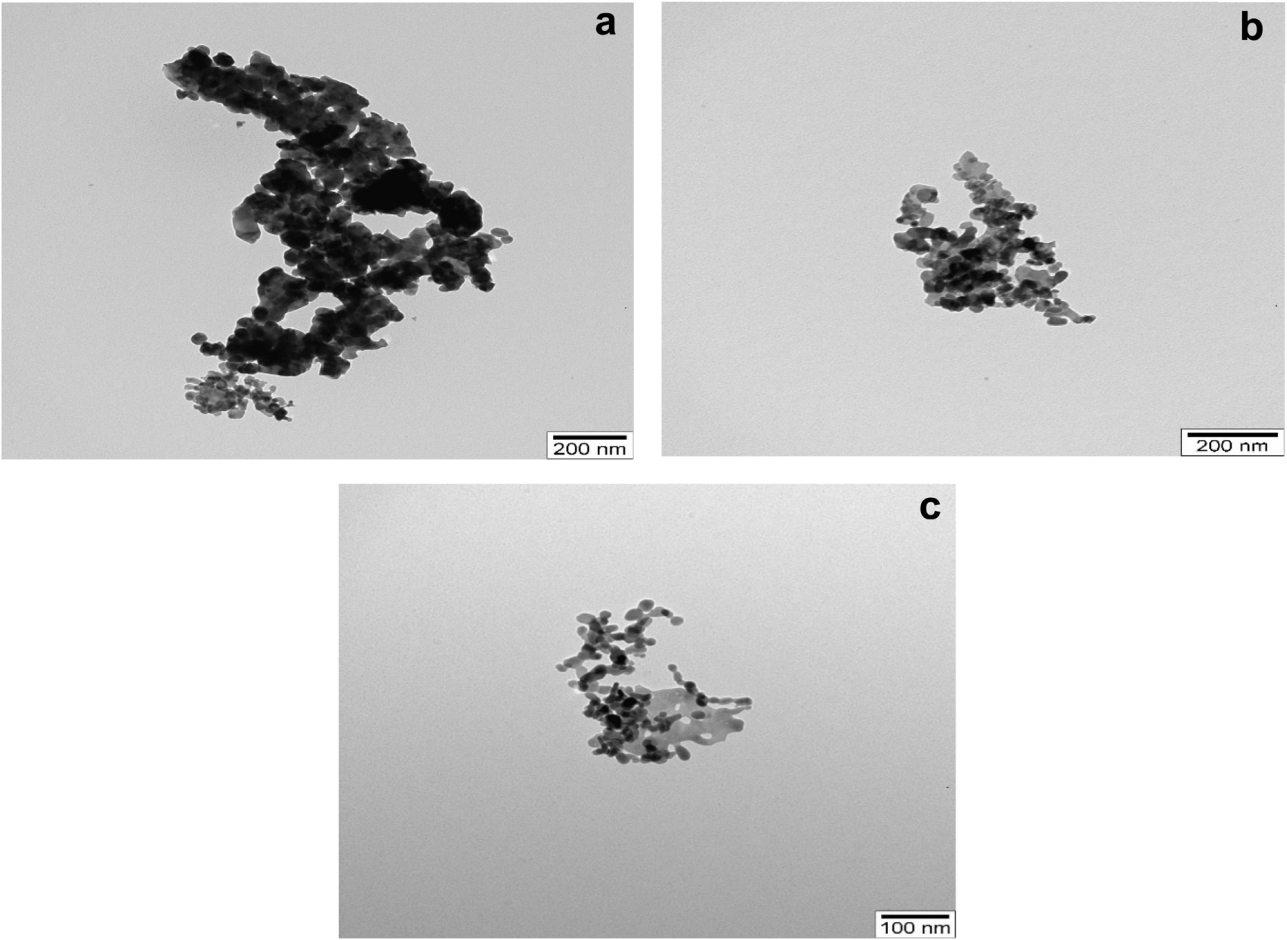
TEM micrograph of Co_3_O_4_ nanoparticles: (a) Co_3_O_4_-HT, (b) Co_3_O_4_-SC, and (c) Co_3_O_4_-CP.

Fig. 8 presents the TEM images of (a) Co_3_O_4_-HT, (b) Co_3_O_4_-SC, and (c) Co_3_O_4_-CP samples. The TEM analysis reveals that the synthesis method plays a decisive role in the formation of Co_3_O_4_ nanoparticles. The Co_3_O_4_-HT sample shown in Fig. 8a is characterized by an ordered growth resulting from hydrothermal conditions. In the hydrothermal environment, the coordination sphere of Co^2+^ ions promotes the preferential formation of low-energy crystallographic planes (*e.g.*, (111) and (100)). The structures observed at the 200 nm scale typically exhibit polyhedral morphologies, which tend to grow further and stabilize with prolonged hydrothermal duration. The relatively aggregated structures observed in micrograph (a) reflect the tendency of particles in hydrothermal systems to collide and form larger clusters with lower surface energy. During the hydrothermal process, the solubility of precursors (*e.g.*, cobalt nitrate) increases, providing favorable conditions for homogeneous nucleation. The morphology observed in (a) can be explained by the Ostwald ripening mechanism. In contrast, the Co_3_O_4_-SC sample shown in Fig. 8b consists of smaller and more uniformly distributed nanoparticles compared to the hydrothermal sample. As seen in micrograph (b), the particles exhibit predominantly spherical or quasi-spherical shapes. This is attributed to the extremely rapid cooling (>10^9^ K s^−1^) following the collapse of cavitation bubbles. Such rapid quenching prevents the full development of low-energy crystal facets and kinetically “freezes” the particles in a spherical morphology. Furthermore, the presence of weakly connected clusters or interparticle separation in (b) arises from the combined effects of sonochemical energy and the stabilizer (PVA). Finally, the Co_3_O_4_-CP sample shown in Fig. 8c, presented at a 100 nm scale, exhibits a highly aggregated and porous structure. As observed, individual nanoparticles undergo coalescence during the thermal decomposition of the precursor, forming chain-like aggregates with voids within the clusters. This clustering behavior observed in TEM is directly influenced by the calcination temperature and duration.

**Fig. 9:**
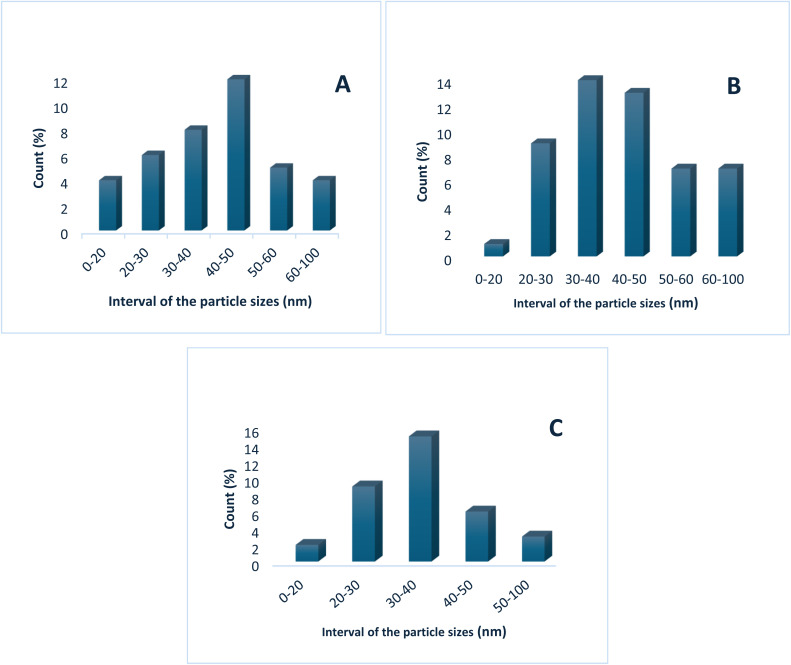
Particle size distribution of (A) Co_3_O_4_-HT, (B) Co_3_O-SC, and (C) Co_3_O_4_-CP nanoparticle samples derived from TEM analysis.

Fig. 9 presents histograms (A, B, C) derived from TEM images revealing the size characteristics of the synthesized Co_3_O_4_ samples and their correlation with the Williamson–Hall (W–H) results obtained from XRD analysis. Histogram (A) shows that the Co_3_O_4_-HT sample exhibits a relatively broad size distribution, with centered around approximately 40–50 nm. The crystallite size calculated by the W–H method (33.6 nm) suggests that the particles observed in TEM may consist of several smaller crystallites, or that microstrain present in the surface layers contributes to the observed size discrepancy. Histogram (B) for the Co_3_O_4_-SC sample displays a sharper peak, with the majority of particles concentrated around 30–40 nm. This result is in excellent agreement with the crystallite size obtained from the W–H method (28 nm). Such close correspondence indicates that, during sonochemical synthesis, each particle is composed of nearly a single crystal and possesses high structural quality. Histogram (C) of the Co_3_O_4_-CP sample shows particle diameters centered around ∼35 nm, whereas the W–H method gives a much smaller crystallite size of 12.5 nm. This discrepancy indicates that the ∼35 nm particles observed in TEM are actually porous aggregates formed by the agglomeration of numerous ultra-small primary crystallites (∼12.5 nm).

### 3.6. UV-Vis absorption

**Fig. 10:**
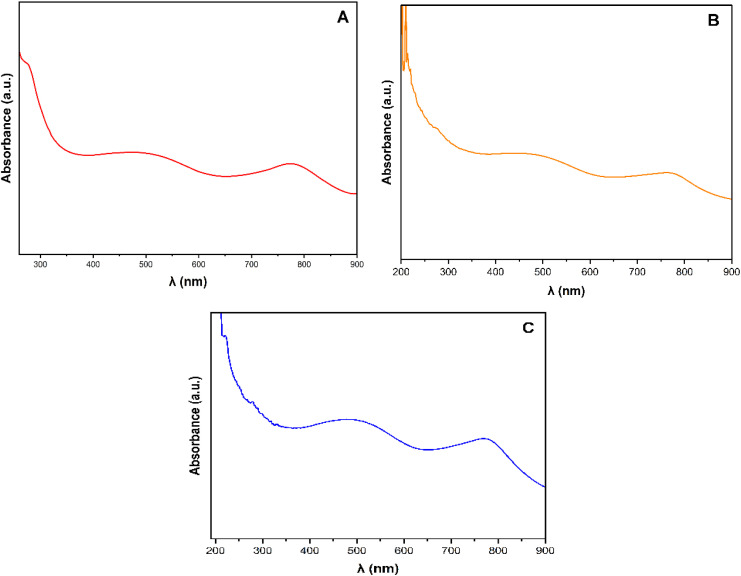
Optical absorption behavior of (A) Co_3_O_4_-HT, (B) Co_3_O_4_-SC, and (C) Co_3_O_4_-CP nanoparticle samples in the UV-visible region.

Fig. 10A–C illustrates the UV-visible absorption spectra of the Co_3_O_4_ nanoparticles synthesized *via* hydrotherm Co_3_O_4_-HT (A), Co_3_O_4_-SC (B), and Co_3_O_4_-CP (C) methods, respectively. All samples exhibit a strong absorption band in the ultraviolet region below 350 nm, which can be attributed to charge–transfer transitions between O^2−^ (2p) and Co^3+^ (3d) orbitals, a characteristic feature of spinel-type Co_3_O_4_ structures. This observation confirms the formation of the Co_3_O_4_ phase with a normal spinel configuration. A noticeable broad absorption tail extending into the visible region (400–800 nm) suggests the presence of ligand-to-metal charge transfer (LMCT) and possible d–d transitions of Co^2+^ and Co^3+^ ions located in tetrahedral and octahedral sites, respectively. These transitions are typically observed near 530–750 nm and are linked to the mixed valence states within the Co_3_O_4_ spinel lattice. Comparatively, the Co_3_O_4_-SC sample (B) demonstrates a slightly higher absorption intensity and a more pronounced tail in the visible region, indicating enhanced light-harvesting ability. This effect can be attributed to the reduction in particle size and enhanced surface defect density introduced by sonochemical processing. The Co_3_O_4_-HT sample (A) shows a smoother absorption edge, reflecting higher crystallinity and larger particle size. Meanwhile, the Co_3_O_4_-CP sample (C) presents an intermediate optical profile with a slightly red-shifted edge, which could indicate the narrowing of the optical band gap due to increased defect levels or sub-band-gap states induced during chemical precipitation. The observed red-shift of the absorption edge from the hydrothermal to the sonochemical samples implies a decrease in the optical band gap energy. This trend aligns with previously reported data for metal-doped and surface-modified Co_3_O_4_ nanoparticles, where smaller crystallite size and oxygen vacancy formation lead to improved optical absorption in the visible region. A comparative analysis of the spectra reveals that sample Co_3_O_4_-CP is characterized by sharper and more well-defined absorption peaks compared to the others. In particular, the pronounced sharpness of the peaks observed at wavelengths of 480 nm and 780 nm indicates a monodisperse particle size distribution and a high degree of crystallinity in this sample. In contrast, the broader and smoother absorption bands observed in samples Co_3_O_4_-HT and Co_3_O_4_-SC suggest a polydisperse size distribution of the particles. The peak broadening (inhomogeneous broadening) can be attributed to the overlap of electronic transitions originating from particles of varying sizes. These findings confirm that the synthesis conditions employed for sample Co_3_O_4_-CP enable more controlled particle size regulation.

**Fig. 11:**
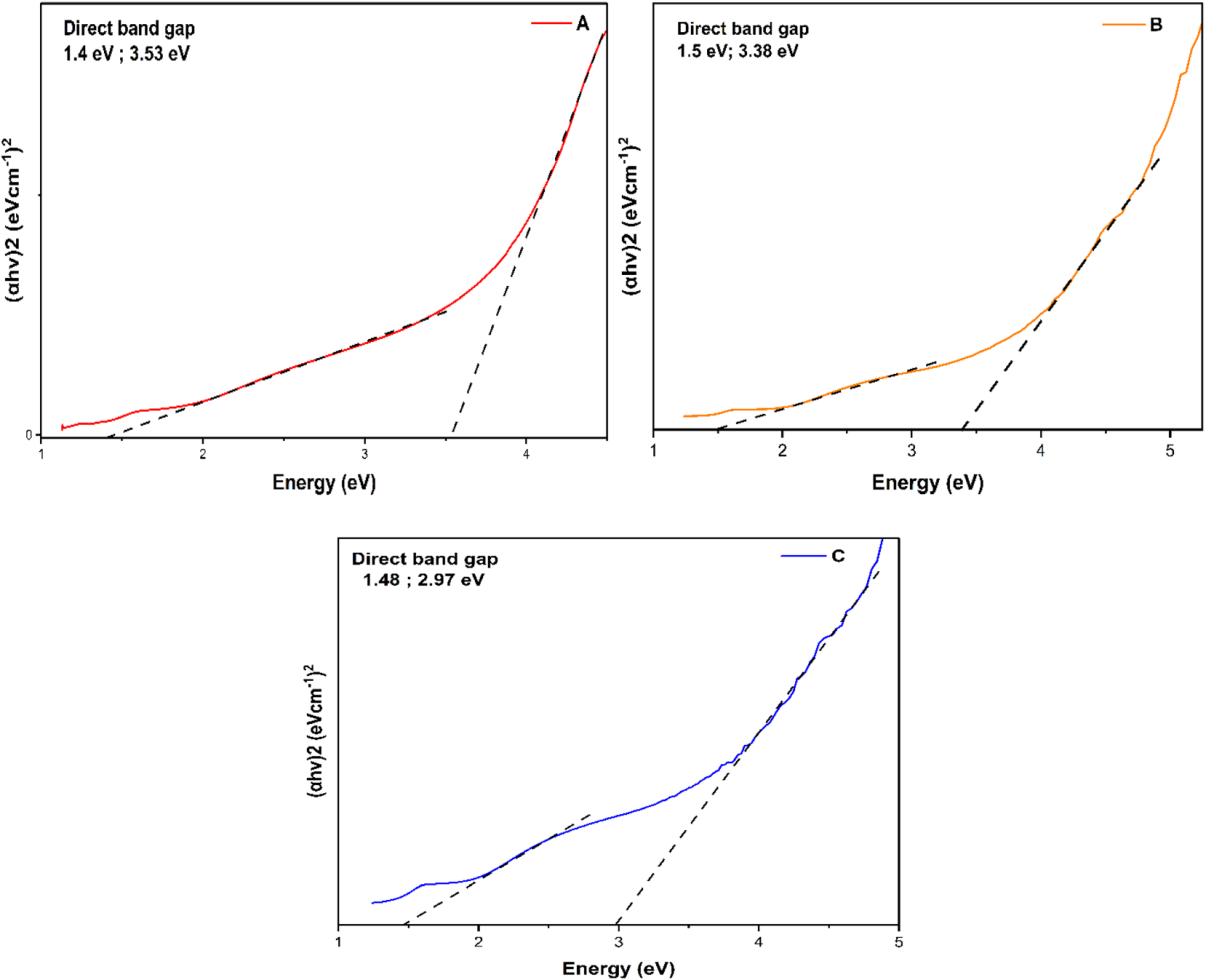
Tauc plots for determination of direct optical band gaps of (A) Co_3_O_4_-HT, (B) Co_3_O_4_-SC, and (C) Co_3_O_4_-CP nanoparticle samples.

The optical band gaps of all three Co_3_O_4_ samples were determined from the absorption spectra shown in Fig. 10 using the Tauc plot methodology, and the results are presented in Fig. 11. This method is based on the mathematical relationship between the absorption coefficient (*α*) and the incident photon energy (*hν*). In general, this relationship can be expressed by the following equation:^52^

7 (*αhν*)^*n*^ = *A*(*hν* − *E*_g_)

*A* is a constant that depends on the structure and type of the sample, *E*_g_ is the band gap, and the value of *n* depends on the nature of the transition: *n* = 2 for direct allowed transition, *n* = 1/2 for indirect allowed transition, *α* is the absorption coefficient, its value is obtained from the Beer–Lambert formula.

The Tauc plot obtained for the Co_3_O_4_-HT sample in Fig. 11A reveals two distinct direct band gaps at 1.4 eV and 3.53 eV. The value of 1.4 eV corresponds to the first band gap typical for Co_3_O_4_ and is close to the bulk material value (∼1.48 eV). However, the second band gap at 3.53 eV is significantly higher than the commonly reported values in the literature (typically 2.1–2.2 eV).^23^ This can be explained by several factors. First, during hydrothermal synthesis, the reduction in particle size may lead to the onset of quantum confinement, resulting in band gap widening. Second, the highly ordered crystal lattice formed under hydrothermal conditions may enhance the splitting of energy levels between tetrahedral and octahedral sites, thereby increasing the transition energies. In Fig. 11B, the direct band gaps determined for the Co_3_O_4_-SC sample are 1.5 eV and 3.38 eV. The value of 1.5 eV may be attributed to the presence of structural defects or oxygen vacancies introduced by the sonochemical method, which lead to a red shift of the absorption edge. In Fig. 11C, the band gap value of 2.97 eV for the Co_3_O_4_-CP sample is lower compared to those of Co_3_O_4_-HT (3.53 eV) and Co_3_O_4_-SC (3.34 eV). This indicates that the quantum confinement effect is less pronounced in this sample.

### 3.7. SEM and EDS analysis

**Fig. 12:**
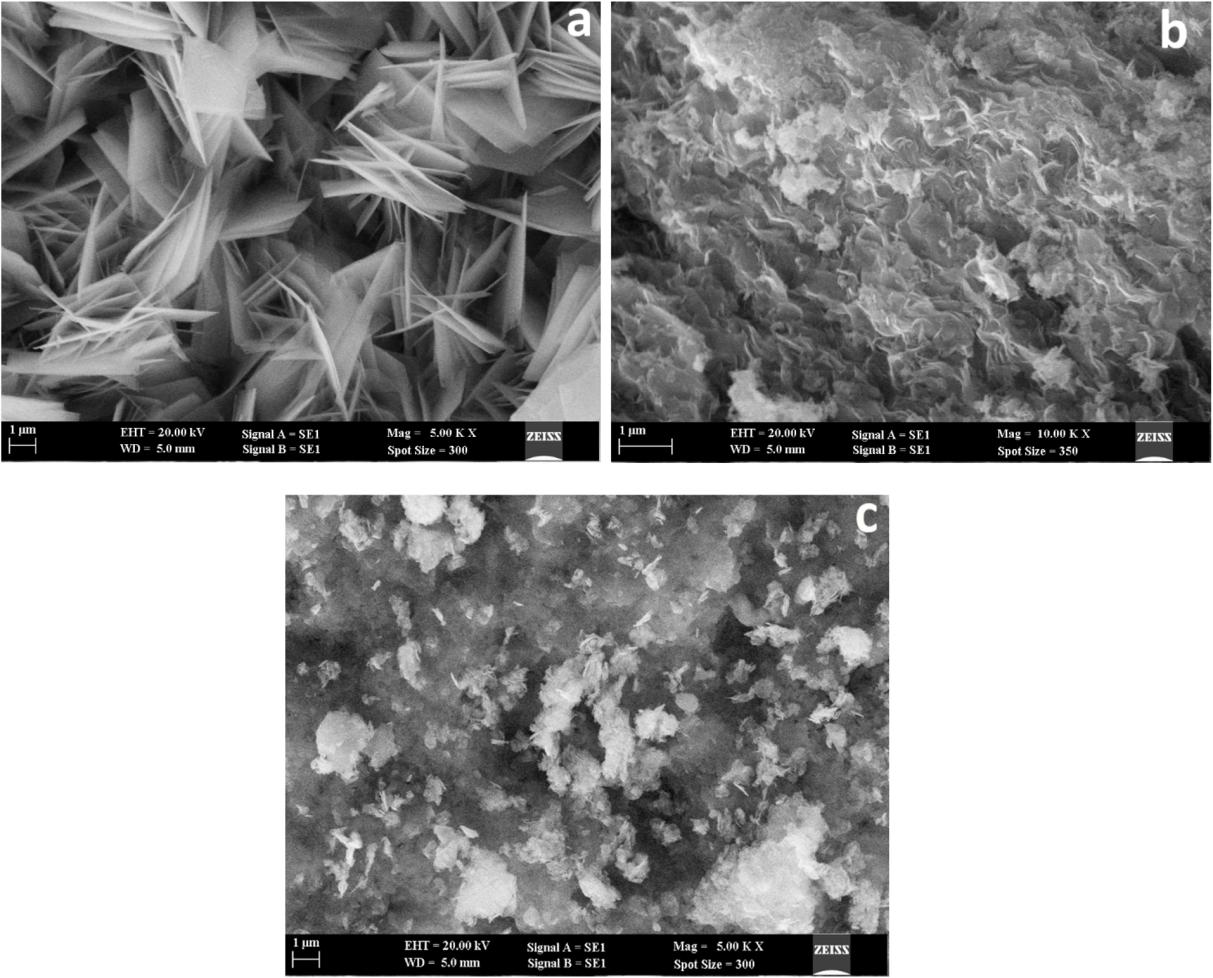
SEM image of the Co_3_O_4_-HT (a), Co_3_O_4_-SC (b), and Co_3_O_4_-CP (c) samples.

The surface morphology of the synthesized Co_3_O_4_ nanostructures was investigated based on the SEM micrographs presented in Fig. 12. A comparative analysis reveals that the energy dynamics of the synthesis environment play a decisive role in determining the growth direction and final morphology of the particles.

The hydrothermally synthesized Co_3_O_4_-HT sample (Fig. 12a) is characterized by highly ordered nanosheet structures with sharp edges and well-developed morphology. The thickness of these nanosheets ranges from approximately 70 to 100 nm, while their length varies between 4 and 5 µm. The interconnection of these plate-like structures leads to the formation of flower-like aggregates. This morphology can be attributed to the preferential crystal growth along specific crystallographic planes under conditions of elevated temperature and autogenous pressure, resulting in high crystallinity and a large specific surface area. In the case of the Co_3_O_4_-SC sample (Fig. 12b), the influence of ultrasonic cavitation results in thinner and more wrinkled structures. The surface appears rougher and more heterogeneous, indicating a non-uniform distribution of active surface sites. The localized high-temperature and high-pressure conditions generated during cavitation contribute to the fragmentation of larger crystals and promote the formation of layered structures with an increased specific surface area. For the Co_3_O_4_-CP sample obtained *via* the chemical precipitation method (Fig. 12c), no well-defined geometry is observed. Instead, a granular morphology dominates, consisting of smaller, irregularly shaped nanoparticles that are densely agglomerated. These morphological features are consistent with the smallest crystallite size (12.1 nm) and the high dislocation density determined from XRD analysis. Although a high degree of dispersion is observed, the distinction of individual particles remains challenging.

Overall, the results demonstrate that the choice of synthesis method has a direct impact on the morphology of Co_3_O_4_ nanostructures, which in turn plays a crucial role in their potential functional applications.

**Fig. 13:**
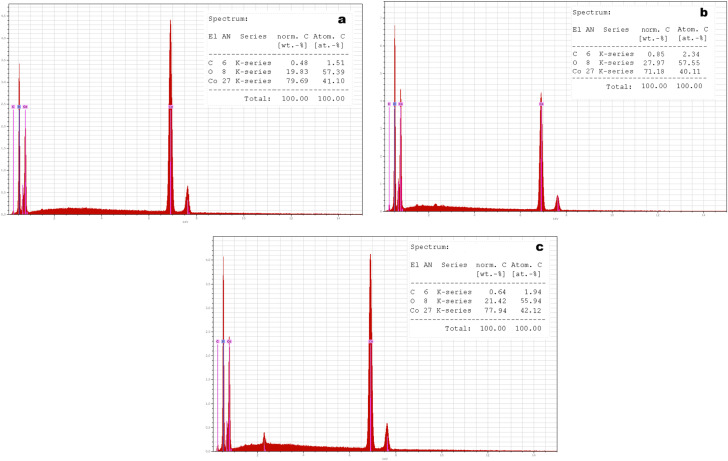
EDS spectrum and elemental quantitative results of the Co_3_O_4_-HT (a), Co_3_O_4_-SC (b), and Co_3_O_4_-CP (c) samples.

The EDS results of the synthesized Co_3_O_4_ nanostructures indicated that the primary elements, *i.e.*, Co and O, are present in all samples, as shown in Fig. 13a–c. In the samples ranging from Co_3_O_4_-HT to Co_3_O_4_-CP, the elemental weight and atomic percentages exhibit slight variations dictated by the specific synthesis route, which is also evident in the EDS spectra. In Co_3_O_4_-HT (Fig. 13a), the atomic percentage of Co is approximately 41.10%, showing a near-stoichiometric ratio suitable for high crystallinity. In Co_3_O_4_-SC (Fig. 13b), the concentration of O is noticeably higher (57.55 at%) compared to the HT sample, which can be attributed to the enhanced oxidation environment induced by acoustic cavitation during the sonochemical process. Furthermore, in Co_3_O_4_-CP (Fig. 13c), the EDS exhibits a balanced weight and atomic ratio (42.12 at% for Co and 55.94 at% for O), confirming the successful formation of the spinel phase *via* chemical precipitation. These results are consistent with the XRD analyses, confirming that the spinel-structured Co_3_O_4_ phase was successfully synthesized in all samples.

## 4 Conclusion

The results of this study demonstrate that the selection of the synthesis method is a crucial factor for the targeted tuning of the structural and optical properties of Co_3_O_4_ nanoparticles. The successful formation of a pure spinel phase with high crystallinity using all three applied methods: hydrothermal, sonochemical, and chemical precipitation confirms the effectiveness of the synthesis procedures. In particular, the dominance of the (311) diffraction peak indicates the development of a preferred crystallographic orientation within the material. The microstructural parameters of the material vary significantly depending on the synthesis environment. The chemical precipitation method produces a defect-dominant structure characterized by the smallest crystallite size (12.5 nm) and a high dislocation density, whereas the hydrothermal method results in the formation of larger crystallites (33.6 nm) with a more ordered polyhedral morphology and a strain-dominant structure. The sonochemical method, in contrast, occupies an intermediate position in terms of both crystallite size (∼28 nm) and structural order, exhibiting a balanced structural configuration. The combined morphological and optical analyses reveal that these structural differences are directly reflected in the electronic properties of the materials. TEM observations confirm the formation of ordered polyhedral (Co_3_O_4_-HT), well-dispersed spherical (Co_3_O_4_-SC), and porous agglomerated (Co_3_O_4_-CP) morphologies, which in turn influence the electronic band structure of the material. In defect-rich Co_3_O_4_-SC and Co_3_O_4_-CP samples, the band gap narrows to approximately ∼1.27 eV, whereas in the highly crystalline Co_3_O_4_-HT sample, it reaches a maximum value of ∼3.53 eV. The SEM analysis demonstrated that the synthesis method critically governs the morphology of Co_3_O_4_ nanostructures, leading to distinct architectures ranging from well-defined nanosheets to agglomerated granular particles. Thus, the study demonstrates a direct correlation between nanoparticle size, internal defect density, optical properties, and morphological variations, showing that these characteristics can be effectively controlled through the choice of synthesis technique. Such an approach is particularly significant for applications where surface reactivity, charge transport, and light–matter interactions play a critical role.

## Author contributions

Fatma Ismayilova: investigation, data curation, formal analysis, writing – original draft, writing – review & editing. Mustafa Muradov: supervision, project administration, conceptualization. Zeynab Addayeva: methodology, formal analysis. Sevinj Mammadyarova: synthesis. Gasimov Eldar: formal analysis, data curation. Rzayev Fuad: methodology, formal analysis, data curation. Orkhan Gulahmadov: methodology, formal analysis, data curation.

## Conflicts of interest

The authors declare that they have no known competing financial interests or personal relationships that could have appeared to influence the work reported in this paper.

## Data Availability

There is no additional data associated with this article.

## References

[cit1] Danish M. S. S., Estrella L. L., Alemaida I. M. A., Lisin A., Moiseev N., Ahmadi M. (2021). *et al.*, Photocatalytic applications of metal oxides for sustainable environmental remediation. Metals.

[cit2] Mansurov Z. A., Hashami M. (2025). Synthesis of Co_3_O_4_ NPs by solution combustion synthesis (SCS) and their structure morphology: a mini review. Combust. Plasma Chem..

[cit3] Danish M. S., Bhattacharya A., Stepanova D., Mikhaylov A., Grilli M. L., Khosravy M., Senjyu T. (2020). A systematic review of metal oxide applications for energy and environmental sustainability. Metals.

[cit4] Araújo E. S., Pereira M. F., da Silva G. M., Tavares G. F., Oliveira C. Y., Faia P. M. (2023). A review on the use of metal oxide-based nanocomposites for the remediation of organics-contaminated water *via* photocatalysis: fundamentals, bibliometric study and recent advances. Toxics.

[cit5] Mashan T. T., Hashami M., Bergeneva N. S., Nurmukhanbetova N. N., Beisebayeva A. S., Nazhipkyzy M. (2025). *et al.*, A comprehensive overview of Co_3_O_4_ nanoparticles: solution combustion synthesis and potential applications. Nanomaterials.

[cit6] Pabba D. P., Satthiyaraju M., Ramasdoss A., Sakthivel P., Chidhambaram N., Dhanabalan S., Thirumurugan A. (2023). MXene-based nanocomposites for piezoelectric and triboelectric energy harvesting applications. Micromachines.

[cit7] Haritha B., Deepak M., Dhananjaya M., Hussain O. M., Julien C. M. (2025). Lanthanum-Doped Co_3_O_4_ Nanocubes Synthesized *via* Hydrothermal Method for High-Performance Supercapacitors. Nanomaterials.

[cit8] Tadesse G., Ananda Murthy H. C., Ravikumar C. R., Naveen Kumar T., Teshome L., Desalegn T. (2023). In Situ Green Synthesis of Co_3_O_4_@ ZnO Core-Shell Nanoparticles Using Datura stramonium Leaf Extract: Antibacterial and Antioxidant Studies. Bioinorg. Chem. Appl..

[cit9] Zaki N. H. M., Mustaffa M., Taib M. F. M., Hassan O. H., Yahya M. Z. A., Ali A. M. M. (2018). Understanding the electronic transition of normal spinel structure of Co_3_O_4_ using GGA+ U calculations. Int. J. Eng. Technol.

[cit10] Sun H., Ang H. M., Tadé M. O., Wang S. (2013). Co_3_O_4_ nanocrystals with predominantly exposed facets: synthesis, environmental and energy applications. J. Mater. Chem. A.

[cit11] El-Shamy O. A., Deyab M. A. (2023). The most popular and effective synthesis processes for Co_3_O_4_ nanoparticles and their benefit in preventing corrosion. Z. Phys. Chem..

[cit12] Wei X., Kang J., Gan L., Wang W., Yang L., Wang D. (2023). *et al.*, Recent advances in Co_3_O_4_ based composites: Synthesis and application in combustion of methane. Nanomaterials.

[cit13] Wang D., Yu Y., He H., Wang J., Zhou W., Abruna H. D. (2015). Template-free synthesis of hollow-structured Co_3_O_4_ nanoparticles as high-performance anodes for lithium-ion batteries. ACS Nano.

[cit14] Matyszczak G., Krawczyk K., Yedzikhanau A., Głuc K., Szymajda M., Sobiech A., Gackowska Z. (2024). Sonochemical Synthesis of Low-Dimensional Nanostructures and Their Applications-A Review. Materials.

[cit15] Dekkiche G., Benabdellah A., Harid N. (2025). The Effect of Polyethylene Glycol on Cobalt Oxide Nanoparticles Prepared Using Sonochemical Synthesis. Sustain. Poly. Energy.

[cit16] Li Z., Dong J., Zhang H., Zhang Y., Wang H., Cui X., Wang Z. (2021). Sonochemical catalysis as a unique strategy for the fabrication of nano-/micro-structured inorganics. Nanoscale Adv..

[cit17] Shah S., Shaikh H., Farrukh S., Malik M. I., Bhagat S. (2021). Sonochemical synthesis of Co_3_O_4_ nanoparticles deposited on GO sheets and their potential application as a nanofiller in MMMs for O_2_/N_2_ separation. RSC Adv..

[cit18] Shin C., Manuel J., Kim D. S., Ryu H. S., Ahn H. J., Ahn J. H. (2012). Structural characterization and electrochemical properties of Co_3_O_4_ anode materials synthesized by a hydrothermal method. Nanoscale Res. Lett..

[cit19] Nassar M. Y. (2013). Size-controlled synthesis of CoCO_3_ and Co_3_O_4_ nanoparticles by free-surfactant hydrothermal method. Mater. Lett..

[cit20] Anele A., Obare S., Wei J. (2022). Recent trends and advances of Co_3_O_4_ nanoparticles in environmental remediation of bacteria in wastewater. Nanomaterials.

[cit21] Nassar M. Y., Ahmed I. S. (2011). Hydrothermal synthesis of cobalt carbonates using different counter ions: an efficient precursor to nano-sized cobalt oxide (Co_3_O_4_). Polyhedron..

[cit22] Sabir F. K., Bekele E. T., Gonfa B. A., Edossa G. D., Adino A. T. (2021). Synthesis of cobalt oxide nanoparticles through chemical and biological pathways for antibacterial activity. Journal of Nanostructures.

[cit23] Dittmer A., da Costa Gouveia T. L., Sivalingam K., DeBeer S., Neese F., Maganas D. (2025). Revisiting the band gap problem in bulk Co_3_O_4_ and its isostructural Zn and Al derivatives through the lens of theoretical spectroscopy. Phys Chem Chem Phys.

[cit24] Kishore P. N., Jeevanandam P. (2013). Synthesis of cobalt oxide nanoparticles *via* homogeneous precipitation using different synthetic conditions. J. Nanosci. Nanotechnol..

[cit25] Muradov M. B., Mammadyarova S. J., Eyvazova G. M., Balayeva O. O., Aliyeva G., Hasanova I. (2024). *et al.*, Synthesis of Cu_x_Co_3−x_O_4_ nanoparticles by a sonochemical method and characterization of structural and optical properties and photocatalytic activity for the degradation of methylene blue. RSC Adv..

[cit26] Lakra R., Kumar R., Thatoi D. N., Sahoo P. K., Soam A. (2021). Synthesis and characterization of cobalt oxide (Co_3_O_4_) nanoparticles. Mater. Today: Proc..

[cit27] Latha K. P., Prema C., Sundar S. M. (2018). Synthesis and characterization of cobalt oxide nanoparticles. J Nanosci Technol.

[cit28] Sabeh S. H., Abd Al-Hassan R. J. (2016). Structural, Morphological and Optical Properties of Co_3_O_4_ Thin Film. Eng. Technol. J..

[cit29] Jagtap S. V., Tale A. S., Thakre S. D. (2017). Synthesis by sol gel method and characterization of Co_3_O_4_ nanoparticles. Int. J. Eng. Res. Appl..

[cit30] Ravina S. G., Dalela S., Kumar S., Nasit M., Singh J. (2024). *et al.*, Study of structural, optical, surface and electrochemical properties of Co_3_O_4_ nanoparticles for energy storage applications. Interactions.

[cit31] Yetim N. K. (2021). Hydrothermal synthesis of Co_3_O_4_ with different morphology: Investigation of magnetic and electrochemical properties. J. Mol. Struct..

[cit32] Muradov M. B., Mammadyarova S. J., Eyvazova G. M., Balayeva O. O., Melikova S. Z., Sadigov N. (2024). *et al.*, Sonochemical synthesis and characterization of structural, optical and dielectric properties of Ag-doped Co_3_O_4_ nanoparticles. J. Cluster Sci..

[cit33] Kim N., Kim S. J., Seo S. H., Lee M. J., Jeong B., Kim H. D. (2025). *et al.*, Polymer-Assisted Synthesis of Co_3_O_4_ Spinel Catalysts with Enhanced Surface Co^2+^ Ions for N_2_O Decomposition. Nanomaterials.

[cit34] Li M. T., Fu Q. S., Yang R. S., Wang H. (2014). Co_3_O_4_ nanoparticles prepared by precipitation. Adv. Mater. Res..

[cit35] Hu M., Yang W., Tan H., Jin L., Zhang L., Kerns P. (2020). *et al.*, Template-free synthesis of mesoporous and crystalline transition metal oxide nanoplates with abundant surface defects. Matter.

[cit36] Diallo A., Beye A. C., Doyle T. B., Park E., Maaza M. (2015). Green synthesis of Co_3_O_4_ nanoparticles *via Aspalathus linearis*: physical properties. Green Chem. Lett. Rev..

[cit37] Rogers K. D., Daniels P. (2002). An X-ray diffraction study of the effects of heat treatment on bone mineral microstructure. Biomaterials.

[cit38] Babu C. R., Avani A. V., Xavier T. S., Tomy M., Shaji S., Anila E. I. (2024). Symmetric supercapacitor based on Co_3_O_4_ nanoparticles with an improved specific capacitance and energy density. J. Energy Storage.

[cit39] Bagheri-Mohagheghi M. M., Shahtahmasebi N. M., Alinejad M. R., Youssefi A., Shokooh-Saremi M. (2008). The effect of the post-annealing temperature on the nano-structure and energy band gap of SnO_2_ semiconducting oxide nano-particles synthesized by polymerizing–complexing sol–gel method. Phys. Rev. B: Condens. Matter.

[cit40] Tsaviv J. N., Eneji I. S., Sha’Ato R., Ahemen I., Jubu P. R., Yusof Y. (2024). Photodegradation, kinetics and non-linear error functions of methylene blue dye using SrZrO_3_ perovskite photocatalyst. Heliyon.

[cit41] Jubu P. R., Yam F. K., Chahrour K. M. (2020). Structural and morphological properties of β-Ga_2_O_3_ nanostructures synthesized at various deposition temperatures. Phys. E.

[cit42] Biju V., Sugathan N., Vrinda V., Salini S. L. (2008). Estimation of lattice strain in nanocrystalline silver from X-ray diffraction line broadening. J. Mater. Sci..

[cit43] George K. C., Kurien S., Mathew J. (2007). Lattice strain and lattice expansion of nanoparticles of MgAl_2_O_4_ as a function of particle size. J. Nanosci. Nanotechnol..

[cit44] Lu Y., Weon S., Kim K. H. (2026). From coordination spheres to catalytic sites: Defect engineering in metal oxide photocatalysts. Coord. Chem. Rev..

[cit45] Kurian M., Kunjachan C. (2014). Investigation of size dependency on lattice strain of nanoceria particles synthesised by wet chemical methods. Int. Nano Lett..

[cit46] Andrade A. B., Ferreira N. S., Valerio M. E. (2017). Particle size effects on structural and optical properties of BaF_2_ nanoparticles. RSC Adv..

[cit47] Satar N. A., Aziz A. W., Yaakob M. K., Yahya M. Z. A., Hassan O. H., Kudin T. I. T., Kaus N. M. (2016). Experimental and first-principles investigations of lattice strain effect on electronic and optical properties of biotemplated BiFeO_3_ nanoparticles. J. Phys. Chem. C.

[cit48] Rayapu V. K., Aley R. S., Katlagunta S. S. R., Adiraj S. (2025). The effect of lattice strain on magnetic properties of BaFe_12_O_19_ and NiFe_2_O_4_ thick films prepared *via* tape casting method. Phys. B.

[cit49] Li Z., Sun Y., Ge S., Zhu F., Yin F., Gu L. (2023). *et al.*, An overview of synthesis and structural regulation of magnetic nanomaterials prepared by chemical coprecipitation. Metals.

[cit50] Batlle X., Pérez N., Guardia P. (2011). *et al.*, Magnetic nanoparticles with bulklike properties. J. Appl. Phys..

[cit51] Monshi A., Foroughi M. R., Monshi M. R. (2012). Modified Scherrer equation to estimate more accurately nano-crystallite size using XRD. World J Nano Sci Eng.

[cit52] Suram S. K., Newhouse P. F., Gregoire J. M. (2016). High throughput light absorber discovery, part 1: an algorithm for automated Tauc analysis. ACS Comb. Sci..

